# Time-of-Day-Dependent Post-Induction Hypotension and Personalized Hemodynamic Management in Emergency Spine Surgery: A Retrospective Pre–Post Cohort Study

**DOI:** 10.3390/medicina62030473

**Published:** 2026-03-02

**Authors:** Cheol Lee, Eunsung Park, Jina Kim, Kwangjin Lee

**Affiliations:** 1 Department of Anesthesiology and Pain Medicine, Wonkwang University School of Medicine, 895 Muwang-ro, Iksan-si 54538, Jeonbuk-do, Republic of Korea; kj016415@naver.com; 2 Institute of Wonkwang Medical Science, Wonkwang University School of Medicine, 895 Muwang-ro, Iksan-si 54538, Jeonbuk-do, Republic of Korea; 3 Department of Neurosurgery, Wonkwang University School of Medicine Hospital, 895 Muwang-ro, Iksan-si 54538, Jeonbuk-do, Republic of Korea; silverstar0401@gmail.com; 4 Wonkwang University School of Medicine, 895 Muwang-ro, Iksan-si 54538, Jeonbuk-do, Republic of Korea; juliekim01@naver.com

**Keywords:** post-induction hypotension, emergency spine surgery, personalized hemodynamic management, time of day, norepinephrine, blood pressure targets

## Abstract

*Background and Objectives*: Post-induction hypotension (PIH) is common in emergency spine surgery and may vary by time of day. We evaluated whether a personalized hemodynamic management (PHM) bundle was associated with reduced PIH and hypotension burden. *Materials and Methods*: We conducted a single-center retrospective pre–post cohort study of adults undergoing emergency decompressive or stabilizing spine surgery under general anesthesia. The PHM bundle included documentation of an individualized pre-induction MAP target (default 65 mmHg; higher for selected high-risk phenotypes), dynamic assessment of fluid responsiveness, and proactive vasopressor use (norepinephrine initiated at induction in prespecified high-risk patients), with continuous BP trajectory monitoring. PIH was defined as mean arterial pressure (MAP) < 65 mmHg or a ≥30% decrease from pre-induction MAP within 20 min. We used 1:1 propensity score matching (caliper 0.2) and provider-clustered logistic regression in the matched cohort. *Results*: Among 312 eligible patients (usual care *n* = 200; PHM *n* = 112), PIH varied by time of day, with the highest incidence in morning cases (46.2%; *p* = 0.041). After matching, 224 patients (112 per group) were analyzed. PHM was associated with lower PIH (43.8% vs. 33.0%; adjusted odds ratio 0.62; 95% CI: 0.41–0.94; *p* = 0.024). PHM reduced time-weighted average (TWA) MAP below target (5.7 ± 4.2 vs. 3.2 ± 3.6 mmHg; mean difference (MD) −2.3 mmHg; 95% CI −3.3 to −1.3; *p* = 0.001) and area under MAP < 65 mmHg (ratio 0.62; 95% CI 0.50–0.78; *p* < 0.001). Norepinephrine-equivalent dose was higher (Δ + 20 μg; *p* = 0.005) while rescue phenylephrine boluses were fewer (Δ − 1; *p* < 0.001); crystalloid volume was similar (*p* = 0.151). *Conclusions*: In emergency spine surgery, PIH showed time-of-day variation, and PHM implementation was associated with reduced PIH and hypotension burden.

## 1. Introduction

Post-induction hypotension (PIH) is a frequent and clinically important event during general anesthesia, particularly in high-risk emergency operations [1,2,3,4]. In patients with acute spine trauma, PIH may exacerbate spinal cord ischemia, impair organ perfusion, and contribute to postoperative complications such as acute kidney injury (AKI) and cardiovascular events [4,5].

Circadian physiology and “chronoanesthesia” concepts suggest that time of day may influence hemodynamic responses to anesthetic induction. Diurnal variation in sympathetic tone, neurohormonal regulation, endothelial function, and vascular responsiveness could predispose patients to greater blood pressure drops during specific time windows, especially in the early morning [6,7,8,9].

Personalized hemodynamic management (PHM) seeks to tailor blood pressure targets and vasoactive strategies to individual risk profiles, rather than relying on uniform thresholds such as MAP ≥ 65 mmHg [5]. In patients with chronic hypertension or high-level spinal injuries (e.g., at or above T6), higher perfusion targets may be required to sustain spinal cord and end-organ blood flow [10,11]. PHM programs typically combine individualized MAP targets, dynamic assessment of fluid responsiveness, and proactive vasopressor use to reduce hypotension burden while avoiding unnecessary fluid loading [10]. Empirical evidence for time-of-day variation in peri-induction hypotension (PIH) in emergency spine surgery is limited, and it remains unclear whether structured hemodynamic strategies can offset any such vulnerability given that PIH is still common and its determinants are not fully defined in urgent trauma populations.

We implemented a PHM bundle for emergency spine surgery at a tertiary trauma center and retrospectively evaluated its impact in a pre–post design. The primary objective of this study was to assess whether PHM implementation was associated with a lower incidence of PIH and reduced intraoperative hypotension burden in adults undergoing emergency spine surgery. We also examined whether time-of-day strata were associated with PIH risk and whether PHM effects differed across clinical subgroups. Given the observational and pre–post nature of the study, our analyses were designed to be hypothesis-generating rather than confirmatory.

## 2. Materials and Methods

### 2.1. Study Design and Ethics

We conducted a single-center, pragmatic, retrospective cohort study with an embedded pre–post implementation analysis of a Personalized Hemodynamic Management (PHM) algorithm in adults undergoing emergency spine surgery at a tertiary trauma center. The pre-implementation epoch (usual care) spanned 1 January 2015, to 31 December 2021, and the post-implementation epoch (PHM) spanned 1 January 2022, to 30 September 2025. The PHM bundle was introduced as a local quality-improvement initiative, and this analysis is a retrospective evaluation of routinely collected data. The study protocol was approved by the institutional review board with a waiver of informed consent for minimal-risk retrospective quality-improvement research using de-identified data (WKUH-2025-12-004, approval date: 29 December 2025).

### 2.2. Setting and Participants

We screened all adult patients (≥18 years) who underwent emergency decompression, fixation, or combined procedures for acute traumatic cervical, thoracic, or lumbar spine pathology under general anesthesia. Cases were identified from the electronic health record (EHR) using institutional procedure and diagnosis codes for spine trauma and spine surgery. Operations were performed by spine or trauma surgeons, with anesthetic care provided by board-certified anesthesiologists or supervised residents.

#### 2.2.1. Inclusion and Exclusion Criteria

Eligible patients were adults undergoing emergency spine surgery under general anesthesia with available continuous or 1 min interval blood pressure recordings from ≥5 min before induction through 60 min after induction, and with documented pre-induction MAP (defined as the mean MAP during the final 5 min before induction) and induction agents.

Emergency surgery was defined as an operation performed within 24 h of the decision to operate for acute neurologic compromise or unstable spinal injury. Patients were excluded if they required massive resuscitation before induction (≥4 units of packed red blood cells) or extracorporeal membrane oxygenation (ECMO), because hemodynamics in these settings are dominated by resuscitation rather than anesthetic management. Additional exclusions were pregnancy, transition to comfort-only care before induction, missing minute-level blood pressure data for the primary outcome window, or incomplete records for key covariates.

#### 2.2.2. Time-of-Day and Clinical Strata

Induction time (first administration of induction agent) was used to assign each case to one of four time-of-day strata: morning (07:00–11:59), afternoon (12:00–17:59), evening (18:00–23:59), and night (00:00–06:59). We prespecified clinical strata of interest: chronic hypertension (yes/no, based on medical history or home medication use) and injury level (high-level ≥ T6 vs. lower-level < T6), based on imaging and operative reports.

### 2.3. Anesthetic Management

Induction was typically performed with propofol, opioid (e.g., fentanyl or remifentanil), and neuromuscular blockade, with adjustments for hemodynamic status and comorbidities. Standard monitoring included electrocardiography, pulse oximetry, capnography, and temperature monitoring. Invasive arterial pressure monitoring was used in most cases and was encouraged to be established before induction when feasible; otherwise, non-invasive blood pressure (NIBP) was recorded at 1 min intervals. Ventilation followed lung-protective practices and was adjusted to maintain normocapnia.

#### PHM Bundle and Individualized MAP Targets

The post-implementation “personalized hemodynamic management (PHM)” bundle, introduced in January 2022, consisted of (1) individualized pre-induction MAP targets based on patient-specific risk factors, (2) dynamic assessment of fluid responsiveness, (3) early, proactive vasopressor use in prespecified high-risk phenotypes, and (4) continuous monitoring and documentation of blood pressure trajectories. The primary exposure was care during the pre-implementation versus post-implementation epoch, analyzed using an epoch-level intention-to-treat approach. Because adherence to all PHM components was incomplete after implementation, we conducted a prespecified per-protocol sensitivity analysis restricted to patients with documented individualized MAP targets and pre-emptive norepinephrine administration. Targets were set by the attending anesthesiologist based on baseline blood pressure patterns and comorbidities (e.g., chronic hypertension, cerebrovascular disease). In the post-implementation epoch, targets were routinely documented in the anesthesia record, whereas explicit documentation was uncommon in the pre-implementation epoch. For analyses requiring an individualized target, when no target was recorded, we assigned a default target MAP of 65 mmHg [3], reflecting typical practice before implementation. Patients with chronic hypertension or suspected increased spinal cord perfusion requirements often received higher targets (e.g., 75–85 mmHg) [11].

In the PHM epoch, fluid responsiveness was assessed using pulse pressure variation or stroke volume variation when applicable, or with mini-fluid challenges. Norepinephrine infusions were initiated at induction in predefined high-risk patients (e.g., older age, chronic hypertension, high-level injury) and titrated to maintain the individualized target. Phenylephrine or ephedrine boluses were used for transient hypotension.

### 2.4. Outcomes

The primary outcome was post-induction hypotension (PIH), defined as either a mean arterial pressure (MAP) < 65 mmHg or a ≥30% decrease from pre-induction MAP within the first 20 min after induction. This composite definition reflects both an absolute threshold commonly associated with organ hypoperfusion and a relative decrease that may be critical for patients with chronic hypertension. We selected the 20 min window because this period captures the hemodynamic impact of induction, positioning, and initial surgical exposure, which are potentially modifiable by anticipatory PHM strategies.

Secondary hemodynamic outcomes included time-weighted average (TWA) MAP below the individualized target during the first 60 min after induction, area under MAP < 65 mmHg and under the individualized target (mmHg × min) over 0–60 min, total norepinephrine-equivalent dose, number of rescue vasopressor boluses and crystalloid volume administered in the first 60 min.

Clinical outcomes were AKI within 72 h (kidney disease: Improving Global Outcomes creatinine criteria) and major adverse cardiovascular events (MACE) within 30 days (myocardial infarction, stroke, heart failure, or cardiac death), based on laboratory data and clinical documentation.

#### Data Collection and Missing Data

Minute-level arterial or NIBP data were extracted from the anesthesia information management system and cleaned for obvious artifacts. Clinical, demographic, and outcome variables were obtained from the EHR and trauma registry.

Missing data were <10% for all covariates, mainly for preoperative laboratory variables and intraoperative fluid volumes. We used multiple imputation by chained equations (five imputed datasets) to impute missing covariates, including all variables in the primary analysis models and key outcomes. The exposure (pre- vs. post-implementation epoch) and the primary outcome (PIH) were not imputed. Estimates from the imputed datasets were combined using Rubin’s rules.

### 2.5. Statistical Analysis

We summarized continuous variables as mean ± standard deviation or median (interquartile range, IQR), as appropriate, and categorical variables as counts (percentages). Group comparisons before matching used *t*-tests or Wilcoxon rank-sum tests for continuous variables and chi-square or Fisher’s exact tests for categorical variables.

Propensity scores for being in the PHM epoch were estimated using logistic regression including age, sex, body mass index, chronic hypertension, injury level, baseline MAP, arterial line use at induction, induction agent/dose, time-of-day strata, and year of surgery.

We used 1:1 nearest-neighbor propensity score matching with a caliper of 0.2 standard deviations of the logit of the propensity score to create well-balanced pre- and post-implementation groups for descriptive and primary outcome analyses. We selected PSM, rather than inverse probability weighting, to avoid assigning extreme weights in this modest-sized cohort. Covariate balance after matching was assessed using standardized mean differences (SMDs), with values < 0.1 considered acceptable, and detailed balance diagnostics are reported in Supplementary Table S1 and Supplementary Figure S1.

For the primary outcome, we fit multivariable logistic regression models with robust standard errors clustered by provider, where “provider” was defined as the attending anesthesiologist responsible for intraoperative management. In sensitivity analyses, we fit analogous mixed-effects logistic models with provider-level random intercepts, which yielded similar estimates (Supplementary Table S6).

The primary analysis used multivariable logistic regression for PIH with adjustment for prespecified confounders in the matched cohort. We conducted three prespecified sensitivity analyses: (1) alternative PIH thresholds, (2) analyses in the unmatched cohort with multivariable adjustment, and (3) analyses based on multiple imputation for missing covariates. Additional exploratory analyses, including time-of-day spline models, mediation models, and interrupted time-series/difference-in-differences approaches, are described in the Supplementary Methods and presented as hypothesis-generating, with their main numerical results summarized in Supplementary Tables S2–S6 and Supplementary Figures S2–S4.

Given the modest number of clinical events, we prioritized a limited set of prespecified analyses in the main text and treated additional models (e.g., mediation, interrupted time-series, difference-in-differences) as exploratory, presenting details in the Supplementary Materials. We did not adjust for multiple comparisons, and the results of secondary and subgroup analyses should be interpreted with caution.

Two-sided *p* values < 0.05 were considered statistically significant. Statistical analyses were performed using R (version 4.3.2; R Foundation for Statistical Computing, Vienna, Austria) or equivalent statistical software.

## 3. Results

### 3.1. Patient Flow and Baseline Characteristics

**Figure 1:**
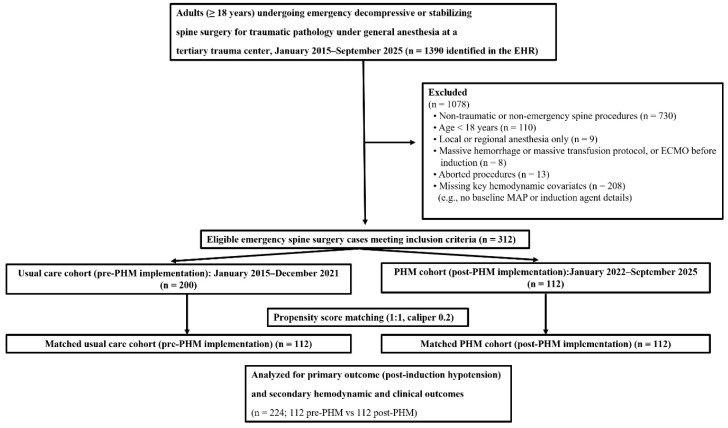
Study flow diagram. personalized hemodynamic management (PHM); propensity score matching (PSM); extracorporeal membrane oxygenation (ECMO); mean arterial pressure (MAP); electronic health record (EHR).

During the study period, 1390 adults undergoing emergency decompressive or stabilizing spine surgery for traumatic pathology under general anesthesia were identified in the electronic health record. Of these, 1078 were excluded because of non-traumatic or non-emergency procedures, age < 18 years, non-general anesthesia, massive hemorrhage or activation of a massive transfusion protocol or extracorporeal membrane oxygenation before induction, aborted procedures, or missing key hemodynamic covariates, leaving 312 eligible cases. These patients were allocated to a usual-care cohort in the pre-PHM epoch (*n* = 200) and a PHM cohort in the post-implementation epoch (*n* = 112), and after 1:1 propensity score matching with a caliper of 0.2, 112 patients from each epoch (total *n* = 224) were included in the analyses of the primary and secondary outcomes (Figure 1).

Before PSM, PHM patients were somewhat younger and less likely to have chronic hypertension, and they had slightly higher baseline MAP and more frequent arterial line use compared with usual care. After 1:1 PSM, 224 patients (112 per group) were retained. After matching, all prespecified covariates had SMDs < 0.10 (Supplementary Table S1 and Supplementary Figure S1), indicating good balance between pre- and post-implementation groups. Baseline characteristics in the matched cohort are summarized in Table 1.

### 3.2. Primary Outcome: Post-Induction Hypotension

In the unmatched cohort, PIH occurred in 44.0% of usual care patients and 33.0% of PHM patients (unadjusted odds ratio (OR): 0.63; 95% CI: 0.40–0.99; *p* = 0.046). PIH incidence varied by time of day, with the highest rates in morning cases (morning 46.2%, afternoon 32.7%, evening 34.4%, night 37.0%; *p* = 0.041). Detailed unmatched hemodynamic and clinical outcomes are presented in Supplementary Table S3.

In the matched cohort, PIH occurred in 49/112 (43.8%) usual care patients and 37/112 (33.0%) PHM patients. In provider-clustered logistic regression models, PHM was associated with lower odds of PIH (adjusted OR: 0.62; 95% CI: 0.41–0.94; *p* = 0.024; Table 2). Morning versus afternoon induction was associated with higher PIH risk, whereas evening and night differences were less pronounced (Supplementary Table S6). Sensitivity analyses using mixed-effects logistic models with provider-level random intercepts yielded estimates that were similar in magnitude and direction to the primary clustered logistic regression models (Supplementary Table S6).

### 3.3. Secondary Hemodynamic Outcomes

In the matched cohort, PHM was associated with reduced hypotension burden (Table 2). TWA MAP below the individualized target over 0–60 min decreased from 5.7 ± 4.2 mmHg (usual care) to 3.2 ± 3.6 mmHg (PHM), with a mean difference (MD) of −2.3 mmHg (95% CI: −3.3 to −1.3; *p* = 0.001). Area under MAP < 65 mmHg also decreased substantially (median 130 [60–245] vs. 75 [32–158] mmHg × min; ratio 0.62; 95% CI: 0.50–0.78; *p* < 0.001). Norepinephrine-equivalent dose was higher in the PHM group (difference: +20 μg; 95% CI: +7 to +33; *p* = 0.005), whereas rescue phenylephrine bolus use decreased (difference: −1 bolus; 95% CI: −2 to −1; *p* < 0.001). Crystalloid volume in the first 60 min was similar between groups (difference: −100 mL; 95% CI: −220 to +20; *p* = 0.151).

**Figure 2:**
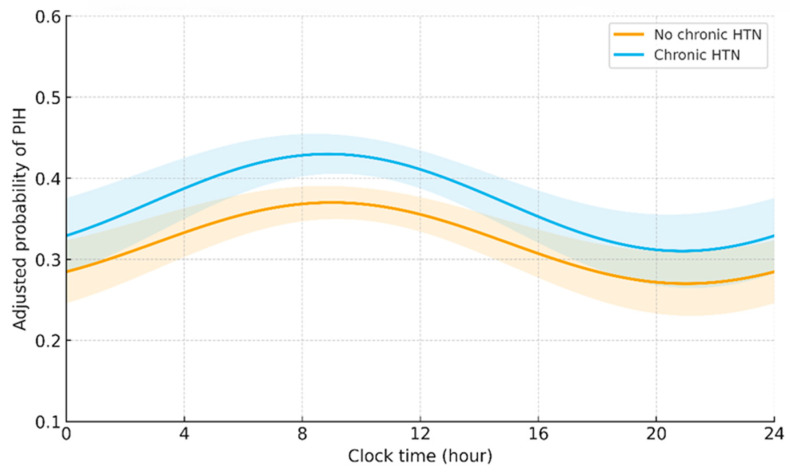
Adjusted probability of PIH across 24 h (time-of-day on x-axis), with separate curves for usual care and PHM, and 95% confidence intervals.

Time-of-day analyses indicated that morning cases had higher TWA MAP deficits and greater AU < 65 mmHg than afternoon cases, with intermediate values in evening and night; these patterns were attenuated but not abolished in the PHM epoch (Figure 2, Supplementary Table S2 and Supplementary Figure S2).

### 3.4. Clinical Outcomes and Implementation Fidelity

In the matched cohort, AKI within 72 h occurred in 11.6% of usual care patients and 7.1% of PHM patients (adjusted OR: 0.59; 95% CI: 0.30–1.12; *p* = 0.110; Table 3). MACE within 30 days was 7.1% versus 5.4% (adjusted OR: 0.78; 95% CI: 0.35–1.72; *p* = 0.540; Table 3). Intensive care unit (ICU) admission and ICU/hospital length of stay showed numerical but not statistically significant improvements with PHM (ICU admission 66.1% vs. 57.1%; adjusted OR: 0.80; 95% CI: 0.53–1.20; *p* = 0.280; ICU length of stay 2.7 [1.5–4.4] vs. 2.3 [1.2–4.1] days; *p* = 0.060; hospital length of stay 9.0 [6.1–13.2] vs. 8.4 [5.7–12.5] days; *p* = 0.200; Table 3).

Implementation fidelity improved after PHM rollout: documentation of individualized MAP targets increased from 19.6% to 90.2% (*p* < 0.001), and pre-emptive norepinephrine use in high-risk patients increased from 10% to 65% (*p* < 0.001; Table 3). Vasopressor-related adverse events were rare (0 vs. 1 case, *p* = 0.320; Table 3).

In a per-protocol sensitivity analysis restricted to post-implementation patients with documented individualized MAP targets and pre-emptive norepinephrine, the association between PHM implementation and reduced post-induction hypotension was directionally similar and numerically larger, although confidence intervals were wider because of the smaller sample size (Supplementary Table S4).

### 3.5. Exploratory Analysis

**Figure 3:**
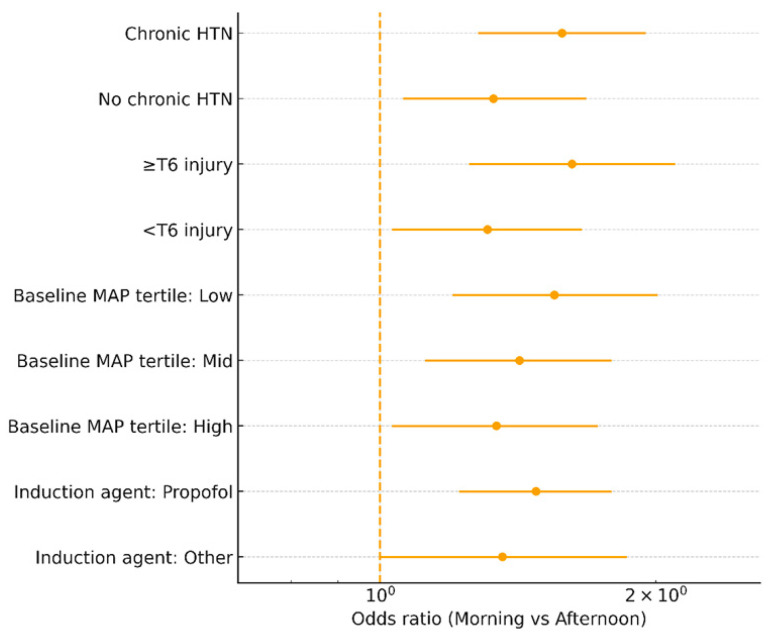
Forest plot of time-of-day (morning vs afternoon) effects on PIH in prespecified subgroups (chronic hypertension, injury level ≥ T6, baseline MAP tertiles, induction agent). Solid lines indicate model-based adjusted estimates; shaded bands indicate 95% confidence intervals; dots (if present) indicate observed proportions.

Prespecified effect-modification analyses of time-of-day (morning vs afternoon) across clinical strata (chronic hypertension, injury level ≥ T6, baseline MAP tertiles, and induction agent) are summarized in Figure 3. Exploratory interrupted time-series and difference-in-differences analyses are presented in the Supplementary Materials. In brief, interrupted time-series models suggested an immediate level decrease of approximately 8 percentage points in monthly PIH rates after PHM implementation (*p* ~ 0.020), without a clear slope change over time (Supplementary Figure S3). Mediation analyses indicated that a portion of the observed association between PHM and AKI risk could be explained by reductions in TWA MAP below target (Supplementary Table S5 and Supplementary Figure S4). Negative control analyses and additional sensitivity analyses are detailed in the Supplementary Methods.

## 4. Discussion

Our study demonstrated the association between implementation of a structured personalized hemodynamic management (PHM) bundle and post-induction hypotension (PIH) in adults undergoing emergency spine surgery [12,13]. In a propensity score-matched cohort, PHM was associated with a lower incidence of PIH and reduced overall intraoperative hypotension burden, as reflected by lower time-weighted average MAP below target and smaller area under MAP < 65 mmHg. These hemodynamic improvements were achieved without clinically meaningful increases in fluid administration and with modestly higher norepinephrine use. Taken together, our findings suggest that a pragmatic PHM approach can be implemented in a real-world trauma setting and may improve intraoperative blood pressure stability; however, given the retrospective pre–post design, these results should be interpreted as hypothesis-generating rather than definitive evidence of causality.

The observed association between PHM and reduced PIH is clinically important because PIH has been linked to myocardial injury, acute kidney injury, and neurologic complications in high-risk surgical populations [5,14,15]. In patients with acute spine trauma, avoiding large and sustained drops in MAP is particularly relevant for spinal cord perfusion and secondary injury prevention. By individualizing MAP targets and promoting anticipatory vasopressor use, PHM aims to maintain perfusion pressure during the vulnerable period immediately after induction, when anesthetic-induced vasodilation, changes in sympathetic tone, and positional factors converge. Even if clinical outcome benefits were underpowered in our cohort, the consistent reductions in hypotension burden support PHM as a hemodynamic quality-of-care target and provide a rational basis for larger trials powered for patient-centered endpoints [16]. The second key finding of our study is that time of day appeared to influence the risk of PIH, with morning cases exhibiting higher PIH rates than afternoon cases, and intermediate risks in evening and night cases [6]. At our institution, morning emergency cases are frequently first-start or early add-on cases following overnight admissions and may be affected by handover and resource ramp-up, whereas afternoon cases more often occur after daytime triage/OR allocation with more stable staffing and logistics; therefore, these contrasts likely capture operational factors in addition to circadian physiology. Our exploratory analyses suggested that the distribution of hypotension burden over the first 60 min after induction also varied across time-of-day strata [17]. However, these patterns may partly reflect differences in case mix, scheduling practices, and staffing rather than intrinsic circadian physiology [15,18]. Because our observational design and available covariates did not allow for full adjustment of these system-level factors, the time-of-day findings should be viewed as hypothesis-generating. Nonetheless, they underscore that anesthesiologists may need to anticipate greater hemodynamic vulnerability in certain time windows and consider more proactive strategies—such as earlier vasopressor initiation—in morning emergency spine cases.

Our results align with and extend previous studies on intraoperative hemodynamic optimization. Randomized trials in high-risk noncardiac surgery and complex spine surgery have reported that individualized hemodynamic strategies—often combining dynamic fluid assessment with vasopressor titration—can reduce the incidence and duration of intraoperative hypotension and, in some cases, decrease postoperative complications [16,19,20]. Observational studies have also suggested that chronic hypertension and higher baseline blood pressure shift the lower limit of autoregulation, making relative drops in MAP particularly hazardous [6,15]. Our PHM bundle adopted similar principles, emphasizing individualized targets and early norepinephrine in high-risk phenotypes, and showed comparable reductions in hypotension burden under emergency conditions. In addition, our exploratory time-of-day analyses complement prior literature on diurnal variation in cardiovascular risk, suggesting that anesthesia-induced PIH may exhibit a temporal pattern that warrants further investigation in prospective circadian-focused studies. This study has some limitations. First, as a single-center pre–post observational study, our findings are vulnerable to confounding by secular trends. Over the study period, other aspects of perioperative and ICU care, staff experience, and institutional protocols may have improved independently of the PHM bundle. Although we adjusted for calendar year and explored interrupted time-series models, these approaches cannot fully disentangle the effects of PHM from broader practice evolution. Second, we excluded patients who required massive transfusion or extracorporeal membrane oxygenation before induction, limiting generalizability to the most hemodynamically unstable trauma patients in whom hemorrhagic shock and advanced organ support dominate hemodynamics. Third, individualized MAP targets were not consistently documented in the pre-implementation epoch, and we assumed a default target of 65 mmHg when missing; this may have introduced measurement error in time-weighted and area-under-threshold metrics, potentially attenuating true associations. Fourth, adherence to individual PHM components in the post-implementation epoch was incomplete, and we defined exposure at the epoch level; resulting exposure misclassification likely biased our main estimates toward the null, although per-protocol sensitivity analyses showed directionally similar and numerically larger effects. Fifth, our primary outcome focused on the first 20 min after induction and may have underestimated hypotension later in the case, although we partially addressed this by analyzing intraoperative time-weighted MAP and area under MAP thresholds as secondary outcomes. Finally, we performed multiple secondary, subgroup, and exploratory analyses without formal adjustment for multiplicity, and some significant findings—especially for infrequent clinical endpoints—may represent chance observations; in addition, the PHM bundle required invasive monitoring and early norepinephrine use that may not be available in all settings, and our multiple imputation strategy assumed missing-at-random mechanisms that cannot be fully verified. Additionally, our dataset did not include standardized long-term neurologic recovery or post-discharge outcomes, so we cannot determine whether reduced PIH translates into durable patient-centered benefit; prospective studies with longer follow-up are needed.

## 5. Conclusions

In conclusion, implementation of a structured PHM bundle in emergency spine surgery was associated with reduced PIH and lower overall intraoperative hypotension burden in a propensity-matched cohort, with signals of potential benefit for kidney and cardiovascular outcomes that require confirmation. Time-of-day analyses suggested that morning emergency cases may be particularly prone to PIH, although these exploratory patterns may reflect a combination of physiologic and system-level factors. Future multicenter randomized or stepped-wedge trials are needed to determine whether PHM causally improves patient-centered outcomes and to clarify how best to integrate individualized blood pressure targets and time-of-day awareness into routine anesthetic practice for spine trauma.

## Figures and Tables

**Table 1:** Baseline characteristics after propensity score matching.

Variable	Usual Care (*n* = 112)	PHM (*n* = 112)	SMD
Age, years, median [IQR]	62.0 [50.0–71.0]	61.0 [49.0–70.0]	0.05
Female, *n* (%)	47 (42.0)	45 (40.2)	0.04
Body mass index, kg × m^−2^	25.1 [22.9–27.7]	25.0 [22.8–27.5]	0.03
Chronic hypertension, *n* (%)	54 (48.2)	52 (46.4)	0.04
Injury level ≥ T6, *n* (%)	44 (39.3)	43 (38.4)	0.02
Baseline MAP, mmHg	88.0 ± 13.0	88.4 ± 13.0	0.03
Arterial line at induction, *n* (%)	81 (72.3)	82 (73.2)	0.02
Propofol induction, *n* (%)	87 (77.7)	88 (78.6)	0.02
Propofol dose, mg × kg^−1^	1.7 [1.4–2.1]	1.6 [1.3–2.0]	0.02
Remifentanil proxy, μg × kg^−1^ × min^−1^	0.12 [0.08–0.16]	0.11 [0.07–0.15]	0.02

Body mass index (BMI); interquartile range (IQR); mean arterial pressure (MAP); personalized hemodynamic management (PHM); standardized mean difference (SMD). Data are presented as mean ± SD, median [IQR], or *n* (%), as appropriate. Baseline characteristics are shown for the propensity score–matched cohort. Balance between groups was assessed using the standardized mean difference (SMD); values < 0.10 were considered indicative of good balance.

**Table 2:** Primary and secondary hemodynamic outcomes in the matched cohort.

Outcome	Usual Care (*n* = 112)	PHM (*n* = 112)	Effect Estimate (95% CI)	*p* Value
PIH (0–20 min), *n* (%)	49 (43.8)	37 (33.0)	aOR 0.62 (0.41–0.94)	0.024
TWA MAP below target, mmHg	5.7 ± 4.2	3.2 ± 3.6	MD −2.3 (−3.3 to −1.3)	0.001
AU < 65 mmHg, mmHg × min	130 [60–245]	75 [32–158]	Ratio 0.62 (0.50–0.78)	<0.001
Norepinephrine-equivalent dose, μg	70 [32–132]	94 [50–162]	Difference +20 (+7 to +33)	0.005
Rescue phenylephrine boluses, *n*	3 [1–5]	1 [0–3]	Difference −1 (−2 to −1)	<0.001
Crystalloid volume (0–60 min), mL	1200 [800–1800]	1100 [800–1600]	Difference −100 (−220 to +20)	0.151

Adjusted odds ratio (aOR); area under the curve (AU); confidence interval (CI); interquartile range [QR] mean arterial pressure (MAP); personalized hemodynamic management (PHM); post-induction hypotension (PIH); time-weighted average (TWA). Data are presented as mean ± SD, median [IQR], or *n* (%), as appropriate. Effect estimates are reported with 95% confidence intervals (CI): adjusted odds ratio (aOR) for PIH; absolute between-group difference (Δ) or ratio (as indicated) for continuous outcomes.

**Table 3:** Clinical outcomes and implementation fidelity in the matched cohort.

Outcome/Metric	Usual Care (*n* = 112)	PHM (*n* = 112)	Effect Estimate (95% CI)	*p* Value
AKI within 72 h, *n* (%)	13 (11.6)	8 (7.1)	aOR 0.59 (0.30–1.12)	0.110
MACE within 30 days, *n* (%)	8 (7.1)	6 (5.4)	aOR 0.78 (0.35–1.72)	0.540
ICU admission, *n* (%)	74 (66.1)	64 (57.1)	aOR 0.80 (0.53–1.20)	0.280
ICU length of stay, days	2.7 [1.5–4.4]	2.3 [1.2–4.1]	Difference −0.4 (−0.8 to 0.0)	0.060
Hospital length of stay, days	9.0 [6.1–13.2]	8.4 [5.7–12.5]	Difference −0.6 (−1.5 to 0.3)	0.200
Target MAP documented pre-induction, *n* (%)	22 (19.6)	101 (90.2)	aOR 37.56 (17.26–81.74)	<0.001
Preemptive norepinephrine in high-risk, *n*/*N* (%)	6/60 (10)	39/60 (65)	aOR 16.71 (6.17–45.27)	<0.001
Vasopressor-related adverse events, *n* (%)	0 (0.0)	1 (0.9)	aOR 3.03 (0.12–75.11)	0.320

Acute kidney injury (AKI); adjusted odds ratio (aOR); confidence interval (CI); intensive care unit (ICU); interquartile range (IQR); major adverse cardiovascular events (MACE); mean arterial pressure (MAP); personalized hemodynamic management (PHM). Data are presented as mean ± SD, median [IQR], or *n* (%), as appropriate. Effect estimates are reported with 95% confidence intervals (CI): adjusted odds ratio (aOR) for binary outcomes (approximating crude OR given propensity matching); absolute between-group difference (Δ) for continuous outcomes. High-risk is defined as patients with chronic hypertension or injury level ≥ T6.

## Data Availability

The datasets used and/or analyzed during the current study are available from the corresponding author on reasonable request.

## Supplementary Materials

The following supporting information can be downloaded at: https://www.mdpi.com/article/10.3390/medicina62030473/s1, Table S1: Baseline characteristics before and after propensity score matching; Table S2: Time-of-day-stratified hemodynamic outcomes in the matched cohort; Table S3: Primary and secondary outcomes in the unmatched cohort; Table S4: Per-protocol analysis in high-adherence PHM patients; Table S5: Mediation analysis of the association between PHM and AKI via TWA MAP below target; Table S6: Mixed-effects logistic regression models for PIH with provider-level random intercepts; Figure S1: Propensity score matching balance (Love plot); Figure S2: Adjusted probability of post-induction hypotension across the 24 h clock by epoch; Figure S3: Interrupted time-series analysis of PIH; Figure S4: Conceptual mediation diagram for the association between PHM implementation, hypotension burden, and acute kidney injury Diagram.

## Abbreviations

The following abbreviations are used in this manuscript:

AKI	Acute kidney injury
aOR	Adjusted odds ratio
AU	Area under
BMI	Body mass index
ECMO	Extracorporeal membrane oxygenation
EHR	Electronic health record
ICU	Intensive care unit
IQR	Interquartile range
MACE	Major adverse cardiovascular events
MAP	Mean arterial pressure
MD	Mean difference
NIBP	Non-invasive blood pressure
OR	Odds ratio
PHM	Personalized hemodynamic management
PIH	Post-induction hypotension
SMD	Standardized mean difference
TWA	Time-weighted average
