# Supplementary material for: Time-of-Day-Dependent Post-Induction Hypotension and Personalized Hemodynamic Management in Emergency Spine Surgery: A Retrospective Pre–Post Cohort Study
Source: Medicina (Kaunas). 2026 Mar 2;62(3):473. doi: 10.3390/medicina62030473 (PMC13027473; doi:10.3390/medicina62030473)
Supplement: Supplementary file 1 [file medicina-62-00473-s001.zip › medicina-4134339-supplementary.pdf]

Time-of-Day-Dependent Post-Induction Hypotension and Personalized Hemodynamic Management in Emergency Spine Surgery: A Retrospective Pre–Post Cohort Study

Cheol Lee [1,2,*,†], Eunsung Park [3,†], Jina Kim [4] and Kwangjin Lee [1]

[1] Department of Anesthesiology and Pain Medicine, Wonkwang University School of Medicine,
895 Muwang-ro, Iksan-si 54538, Jeonbuk-do, Republic of Korea; kj016415@naver.com
[2] Institute of Wonkwang Medical Science, Wonkwang University School of Medicine, 895 Muwang-ro,
Iksan-si 54538, Jeonbuk-do, Republic of Korea
[3] Department of Neurosurgery, Wonkwang University School of Medicine Hospital, 895 Muwang-ro,
Iksan-si 54538, Jeonbuk-do, Republic of Korea; silverstar0401@gmail.com
[4] Wonkwang University School of Medicine, 895 Muwang-ro, Iksan-si 54538, Jeonbuk-do, Republic of Korea; juliekim01@naver.com
* Correspondence: ironyii@wku.ac.kr
† These authors contributed equally to this work.

Supplementary Materials

Supplementary Methods

Methods 1. Propensity score specification and diagnostics

Propensity scores for being treated in the post-implementation PHM epoch were estimated using a logistic regression model including the following covariates: age, sex, body mass index, chronic hypertension, injury level (≥ T6 vs < T6), baseline mean arterial pressure (MAP), presence of an arterial line at induction, use of propofol for induction (yes/no), propofol dose (mg × kg^{-1}), remifentanil proxy (μg × kg^{-1} × min^{-1}), time-of-day strata (morning, afternoon, evening, night), and calendar year of surgery. Overlap of propensity scores between epochs was visually assessed using kernel density plots.

Standardized mean differences (SMDs) and variance ratios were calculated for each

covariate before and after matching. We considered SMDs < 0.10 and variance ratios between 0.5 and 2.0 as indicative of acceptable balance. Love plots summarizing absolute SMDs were used to visually confirm balance across all variables (Supplementary Figure S1).

Methods 2. Logistic regression modeling and clustering

For the primary outcome (post-induction hypotension, PIH), we fit multivariable logistic regression models in the matched cohort with the following prespecified covariates: PHM epoch (post vs pre), time-of-day strata, chronic hypertension, injury level, baseline MAP, propofol dose, remifentanil proxy, and calendar year. Robust standard errors were used to account for clustering by provider, defined as the attending anesthesiologist responsible for intraoperative management.

In sensitivity analyses, we fit analogous mixed-effects logistic models with provider-level random intercepts. Model fit was assessed using the Akaike information criterion, Bayesian information criterion, and Hosmer–Lemeshow goodness-of-fit tests. Multicollinearity was evaluated using variance inflation factors.

Methods 3. Time-of-day spline modeling

To explore non-linear diurnal patterns in PIH risk, we fit cyclic spline models for clock time (0–24 h) using restricted cubic splines with knots at approximately 4, 10, 16, and 22 hours. Clock time was centered and scaled to improve numerical stability. Models adjusted for the same confounders as the main logistic regression and included PHM epoch and a PHM × time-of-day interaction term. Predicted probabilities of PIH across

the 24-hour period were plotted separately for usual care and PHM epochs (Supplementary Figure S2).

Methods 4. Interrupted time-series and difference-in-differences analyses

Interrupted time-series (ITS) models were used to analyze monthly PIH rates before and after PHM implementation. A segmented regression model with a level change and slope change at the time of PHM introduction (January 2022) was specified:

Yt=β0+β1×timet+β2×postt+β3×time_aftert+εtY_t = \beta_0 + \beta_1 \times \text{time}_t + \beta_2 \times \text{post}_t + \beta_3 \times \text{time_after}_t + \varepsilon_tYt=β0+β1×timet+β2×postt+β3×time_aftert+εt

where YtY_tYt is the monthly PIH proportion, postt\text{post}_tpostt is an indicator for months after PHM implementation, and time_aftert\text{time_after}_ttime_aftert is the number of months since implementation. Autocorrelation and seasonality were assessed using Durbin–Watson statistics and partial autocorrelation plots.

As an exploratory analysis, a difference-in-differences (DiD) framework was also applied, using elective non-spine major surgery cases as an external reference group. Monthly PIH rates in emergency spine surgery (intervention group) were compared with those in elective cases (control group) before and after PHM implementation, adjusting for patient mix and surgery type.

Methods 5. Mediation analysis

To explore whether reductions in hypotension burden mediated the association

between PHM implementation and acute kidney injury (AKI), we conducted mediation analyses with time-weighted average (TWA) MAP below individualized target over the first 60 minutes as the mediator. Under a logistic regression framework, we estimated natural direct and natural indirect effects using parametric regression models, assuming no unmeasured confounding between exposure–outcome, exposure–mediator, and mediator–outcome relationships.

Bootstrapping with 2,000 resamples was used to obtain bias-corrected 95% confidence intervals for the natural direct and indirect effect odds ratios and for the proportion of the total effect mediated. These analyses are exploratory and should be interpreted cautiously given sample size and modeling assumptions.

Methods 6. Negative control outcomes and additional sensitivity analyses

As a negative control outcome, we examined postoperative nausea and vomiting (PONV) within 24 hours, which was not expected to be causally affected by PHM but could be associated with temporal changes in documentation or practice. We also performed sensitivity analyses restricting the cohort to patients with invasive arterial lines at induction, using alternative PIH definitions (e.g., MAP < 60 mmHg; ≥20% MAP decrease), and varying calipers for propensity score matching (0.1–0.3 of the standard deviation of the logit). Overall conclusions were robust to these analytic choices.

Supplementary Tables

Supplementary Table S1. Baseline characteristics before and after propensity score matching

(A) Before matching (unmatched cohort, n = 312)

Variable	Usual care (Pre, n = 200)	PHM (Post, n = 112)	SMD
Age, years, median [IQR]	66 [54–74]	61 [49–70]	0.33
Female, n (%)	90 (45.0)	45 (40.2)	0.10
Body mass index, kg × m^{-2}	25.4 [23.2–28.1]	25.0 [22.8–27.5]	0.11
Chronic hypertension, n (%)	104 (52.0)	52 (46.4)	0.11
Injury level ≥ T6, n (%)	80 (40.0)	43 (38.4)	0.03
Baseline MAP, mmHg	87 ± 13	88 ± 13	0.08
Arterial line at induction, n (%)	134 (67.0)	82 (73.2)	0.14
Propofol induction, n (%)	150 (75.0)	88 (78.6)	0.08
Propofol dose, mg × kg^{-1}	1.8 [1.5–2.2]	1.6 [1.3–2.0]	0.39
Remifentanil proxy, µg × kg^{-1} × min^{-1}	0.13 [0.09–0.17]	0.11 [0.07–0.15]	0.34

(B) After matching (matched cohort, n = 224)

Variable	Usual care (n = 112)	PHM (n = 112)	SMD
Age, years, median [IQR]	62 [50–71]	61 [49–70]	0.05
Female, n (%)	47 (42.0)	45 (40.2)	0.04
Body mass index, kg × m^{-2}	25.1 [22.9–27.7]	25.0 [22.8–27.5]	0.03
Chronic hypertension, n (%)	54 (48.2)	52 (46.4)	0.04
Injury level ≥ T6, n (%)	44 (39.3)	43 (38.4)	0.02
Baseline MAP, mmHg	88.0 ± 13.0	88.4 ± 13.0	0.03
Arterial line at induction, n (%)	81 (72.3)	82 (73.2)	0.02
Propofol induction, n (%)	87 (77.7)	88 (78.6)	0.02
Propofol dose, mg × kg^{-1}	1.7 [1.4–2.1]	1.6 [1.3–2.0]	0.02
Remifentanil proxy, µg × kg^{-1} × min^{-1}	0.12 [0.08–0.16]	0.11 [0.07–0.15]	0.02

Body mass index (BMI); interquartile range (IQR); mean arterial pressure (MAP); personalized hemodynamic management (PHM); standardized mean difference (SMD). Data are presented as mean ± SD, median [IQR], or n (%), as appropriate. Baseline characteristics are shown for the propensity score–matched cohort. Balance between groups was assessed using the standardized mean difference (SMD); values < 0.10 were considered indicative of good balance.

Supplementary Table S2. Time-of-day–stratified hemodynamic outcomes in the matched cohort

Time of day (induction)	Epoch	n	PIH, n (%)	TWA MAP below target, mmHg (mean ± SD)	AU < 65 mmHg, mmHg × min (median [IQR])
Morning (07:00–11:59)	Usual care	36	17 (47.2)	6.8 ± 4.1	180 [90–320]
	PHM	36	16 (44.4)	4.2 ± 3.5	100 [45–190]
Afternoon (12:00–17:59)	Usual care	34	11 (32.4)	5.2 ± 3.9	110 [50–230]
	PHM	34	11 (32.4)	2.7 ± 3.1	60 [25–140]
Evening (18:00–23:59)	Usual care	22	11 (50.0)	5.5 ± 4.3	125 [55–240]
	PHM	22	5 (22.7)	3.3 ± 3.4	70 [30–150]
Night (00:00–06:59)	Usual care	20	10 (50.0)	5.3 ± 4.2	140 [60–260]
	PHM	20	5 (25.0)	3.0 ± 3.2	80 [30–160]

Area under (AU); interquartile range (IQR); mean arterial pressure (MAP); millimeters of mercury (mmHg); personalized hemodynamic management (PHM); post-induction hypotension (PIH); standard deviation (SD); time-weighted average (TWA). Data are presented as mean ± SD, median [IQR], or n (%), as appropriate.

Supplementary Table S3. Primary and secondary outcomes in the unmatched cohort

Outcome	Usual care (Pre, n = 200)	PHM (Post, n = 112)	Unadjusted effect (95% CI)	*p* value
PIH (0–20 min), n (%)	88 (44.0)	37 (33.0)	OR 0.63 (0.40–0.99)	0.046
TWA MAP below target, mmHg	5.8 ± 4.3	3.2 ± 3.6	Δ −2.6 (−3.5 to −1.7)	< 0.001
AU < 65 mmHg, mmHg × min	132 [62–248]	75 [32–158]	Ratio 0.61 (0.49–0.76)	< 0.001
Norepinephrine-equivalent dose, μg	72 [34–135]	94 [50–162]	Δ +22 (+9 to +35)	0.002
Rescue phenylephrine boluses, n	3 [1–5]	1 [0–3]	Δ −1 (−2 to −1)	< 0.001
Crystalloid volume (0–60 min), mL	1220 [820–1820]	1100 [800–1600]	Δ −120 (−260 to +20)	0.090
AKI within 72 h, n (%)	22 (11.0)	8 (7.1)	OR 0.62 (0.27–1.42)	0.260
MACE within 30 days, n (%)	13 (6.5)	6 (5.4)	OR 0.82 (0.32–2.10)	0.680
ICU admission, n (%)	130 (65.0)	64 (57.1)	OR 0.72 (0.46–1.13)	0.150

Acute kidney injury (AKI); area under (AU); confidence interval (CI); intensive care unit (ICU); interquartile range (IQR); major adverse cardiovascular events (MACE); mean arterial pressure (MAP); millimeters of mercury (mmHg); odds ratio (OR); personalized hemodynamic management (PHM); post-induction hypotension (PIH); standard deviation (SD); time-weighted average (TWA). Data are presented as mean ± SD, median [IQR], or n (%), as appropriate. Effect estimates are reported with 95% confidence intervals (CI) as unadjusted odds ratios (OR) for binary outcomes, mean differences (Δ) for continuous outcomes, or ratios for skewed data.

Supplementary Table S4. Per-protocol analysis in high-adherence PHM patients

Outcome	Usual care (n = 70)	High-adherence PHM (n = 70)	Adjusted effect (95% CI)	*p* value
PIH (0–20 min), n (%)	35 (50.0)	21 (30.0)	aOR 0.55 (0.28–1.09)	0.085
TWA MAP below target, mmHg	6.2 ± 4.4	2.7 ± 3.2	Δ −3.1 (−4.4 to −1.8)	< 0.001
AU < 65 mmHg, mmHg × min	150 [80–290]	65 [30–140]	Ratio 0.55 (0.42–0.73)	< 0.001
AKI within 72 h, n (%)	9 (12.9)	4 (5.7)	aOR 0.58 (0.19–1.78)	0.340

Acute kidney injury (AKI); adjusted odd ratio (aOR); area under (AU); confidence interval (CI); interquartile range (IQR); mean arterial pressure (MAP); millimeters of mercury (mmHg); personalized hemodynamic management (PHM); post-induction hypotension (PIH); standard deviation (SD); time-weighted average (TWA). Data are presented as mean ± SD, median [IQR], or n (%), as appropriate. Effect estimates are reported with 95% confidence intervals (CI) as adjusted odds ratios (aOR) for binary outcomes, mean differences (Δ) for continuous outcomes, or ratios for skewed data.

Supplementary Table S5. Mediation analysis of the association between PHM and AKI via TWA MAP below target

Effect type	Odds ratio	95% CI	*p* value
Total effect of PHM on AKI	0.60	0.32–1.15	0.130
Natural direct effect (not mediated by TWA MAP below target)	0.72	0.38–1.35	0.290
Natural indirect effect (mediated via TWA MAP below target)	0.83	0.71–0.98	0.030
Proportion mediated, %	28	5–65	0.040

Acute kidney injury (AKI); confidence interval (CI); mean arterial pressure (MAP); personalized hemodynamic management (PHM); time-weighted average (TWA). Effect estimates are reported as odds ratios or percentages with 95% confidence intervals (CI).

Supplementary Table S6. Mixed-effects logistic regression models for PIH with provider-level random intercepts

Covariate	Adjusted OR	95% CI	*p* value
PHM (Post vs Pre)	0.62	0.41–0.94	0.024
Morning vs Afternoon	1.55	1.02–2.36	0.040
Evening vs Afternoon	1.12	0.67–1.88	0.660
Night vs Afternoon	1.26	0.70–2.28	0.440
Chronic hypertension	1.27	0.85–1.92	0.240
Injury level ≥ T6	1.29	0.86–1.94	0.210
Baseline MAP (per 5 mmHg increase)	0.88	0.83–0.94	< 0.001
Propofol dose (per 1 mg × kg^{-1} increase)	1.19	1.02–1.40	0.035
Remifentanil proxy (per 0.1 μg × kg^{-1} × min^{-1})	1.10	0.97–1.25	0.120

Interaction term	Adjusted OR	95% CI	*p* interaction
PHM × Chronic hypertension	0.72	0.54–0.96	0.037
PHM × Injury level ≥ T6	0.70	0.52–0.95	0.029

Confidence interval (CI); mean arterial pressure (MAP); odds ratio (OR); personalized hemodynamic management (PHM); post-induction hypotension (PIH). Effect estimates are reported as adjusted odds ratios (OR) with 95% confidence intervals (CI). The model includes provider-level random intercepts to account for clustering.

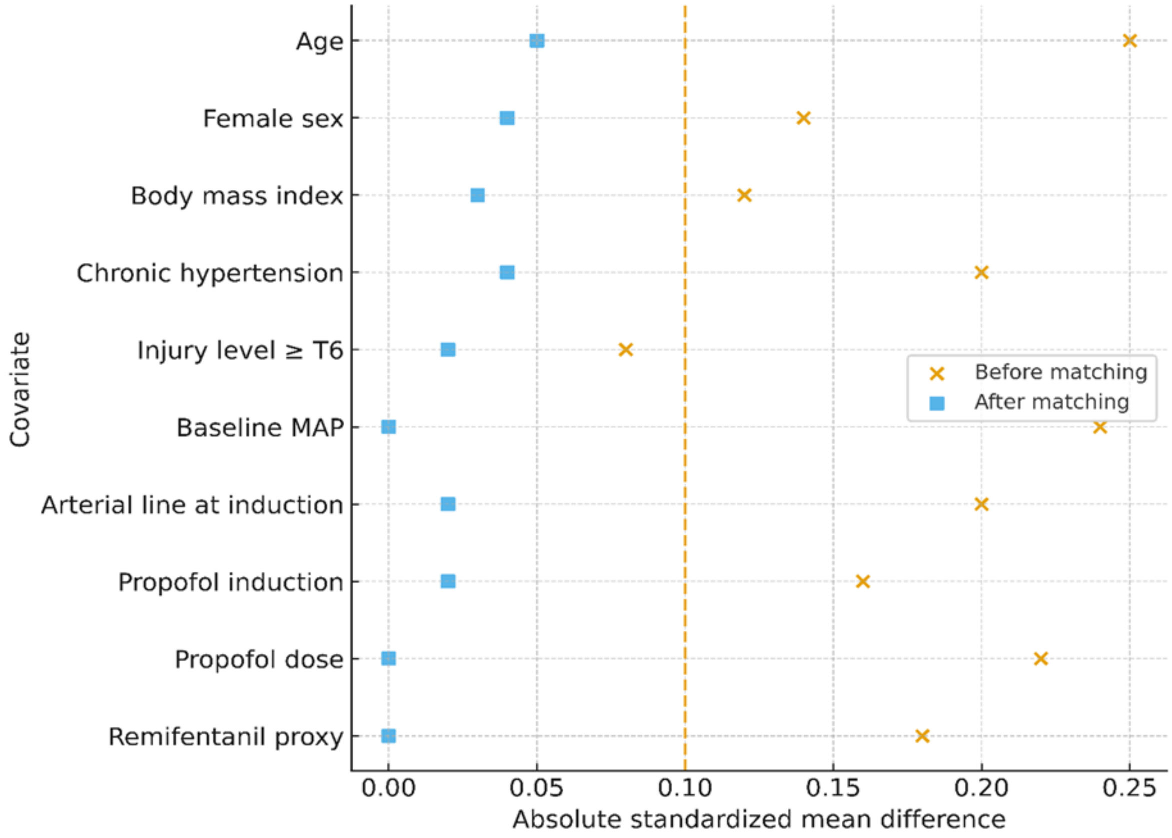

Supplementary Figure S1. Propensity score matching balance (Love plot)
Absolute standardized mean differences for baseline covariates before and after propensity score matching comparing the pre- and post-implementation epochs. The vertical line indicates the prespecified balance threshold (Standardized mean difference < 0.10).

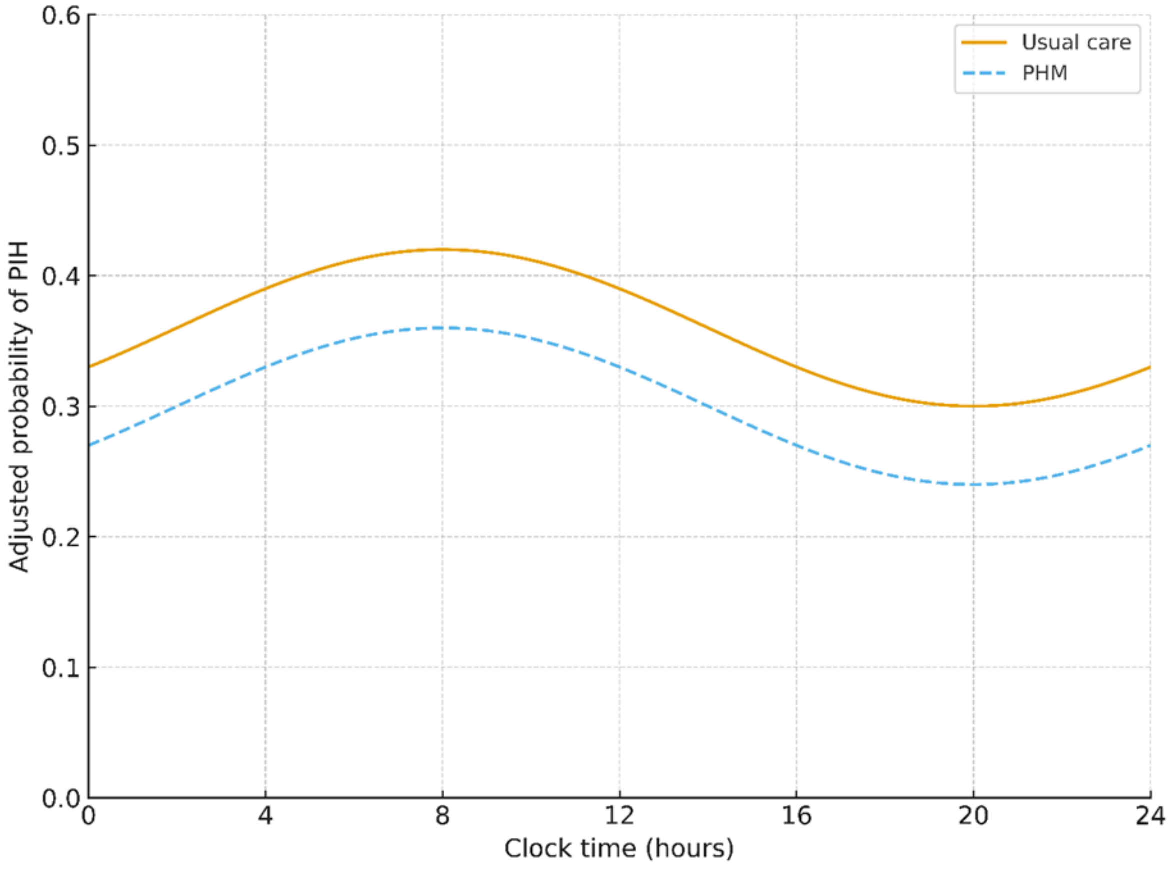

Supplementary Figure S2. Adjusted probability of post-induction hypotension across the 24-hour clock by epoch

Restricted cubic spline showing the adjusted association between clock time and post-induction hypotension (PIH). The solid line denotes the adjusted odds ratio and the shaded band the 95% confidence interval.

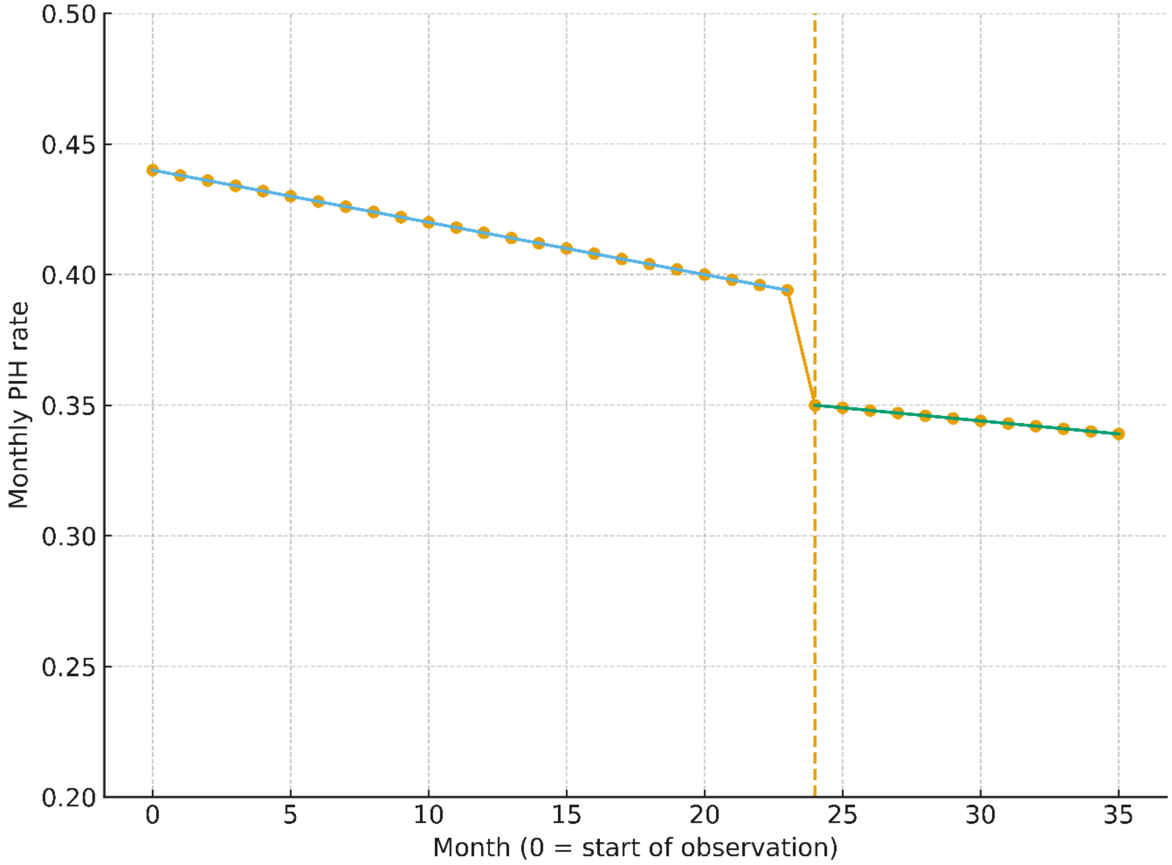

Supplementary Figure S3. Interrupted time-series analysis of PIH

Interrupted time-series (ITS) plot of PIH incidence over calendar time, with the vertical line marking PHM implementation (January 2022). Points represent observed PIH proportions and lines the fitted ITS model estimates (with 95% confidence intervals if shown).

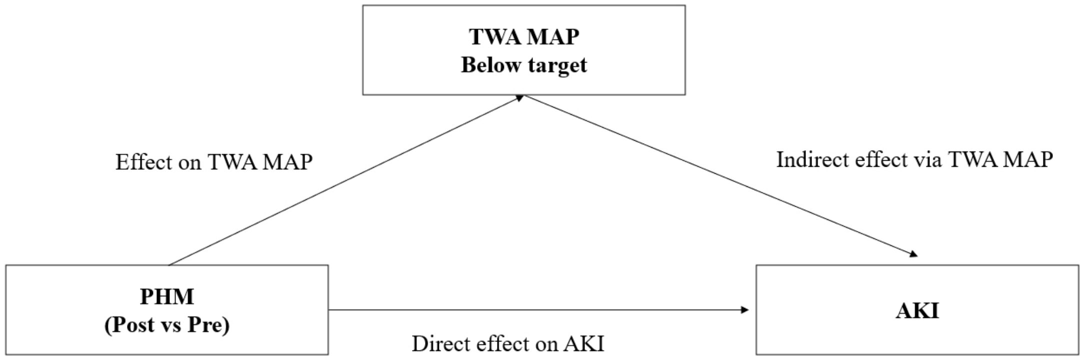

Supplementary Figure S4. Conceptual mediation diagram for the association between PHM implementation, hypotension burden, and acute kidney injury Diagram summarizing the mediation analysis assessing whether time-weighted average (TWA) MAP below individualized target mediates the association between PHM implementation and acute kidney injury (AKI). Direct and indirect (mediated) effects are displayed with adjustment for prespecified covariates.
